# Study of Tissue-Specific Reactive Oxygen Species Formation by Cell Membrane Microarrays for the Characterization of Bioactive Compounds

**DOI:** 10.3390/membranes11120943

**Published:** 2021-11-29

**Authors:** Ane Elexpe, Nerea Nieto, Claudia Fernández-Cuétara, Celtia Domínguez-Fernández, Teresa Morera-Herreras, María Torrecilla, Cristina Miguélez, Antonio Laso, Eneko Ochoa, María Bailen, Azucena González-Coloma, Iñigo Angulo-Barturen, Egoitz Astigarraga, Gabriel Barreda-Gómez

**Affiliations:** 1 Research and Development Division, IMG Pharma Biotech, 48160 Derio, Spain; ane@imgpharma.com (A.E.); nerea.nt@hotmail.com (N.N.); celtia@imgpharma.com (C.D.-F.); egoitz.astigarraga@imgpharma.com (E.A.); 2 Department of Pharmacology, Faculty of Medicine and Nursing, University of the Basque Country UPV/EHU, 48940 Leioa, Spain; teresa.morera@ehu.eus (T.M.-H.); maria.torrecilla@ehu.eus (M.T.); cristina.miguelez@ehu.eus (C.M.); 3 Department of Preventive Medicine and Public Health and Microbiology, Faculty of Medicine, Autonomus University of Madrid UAM, 28029 Madrid, Spain; cuetara96fc@gmail.com (C.F.-C.); maria.bailen@uam.es (M.B.); 4 Neurodegenerative Diseases Group, Biocruces Bizkaia Health Research Instiute, 48903 Barakaldo, Spain; 5 Research and Development Division, AleoVitro, 48160 Derio, Spain; alaso@aleovitro.com (A.L.); eochoa@aleovitro.com (E.O.); 6 Institute of Agricultural Sciences (ICA), Spanish Research Council (CSIC), 28006 Madrid, Spain; azu@ica.csic.es; 7 The Art of Discovery, 48160 Derio, Spain; inigo.ab@tad-med.com

**Keywords:** microarray, inhibitors, antimalarial, superoxide production

## Abstract

The production of reactive oxygen species (ROS) increases considerably in situations of cellular stress, inducing lipid peroxidation and multiple alterations in proteins and nucleic acids. However, sensitivity to oxidative damage varies between organs and tissues depending on the triggering process. Certain drugs used in the treatment of diverse diseases such as malaria have side effects similar to those produced by oxidative damage, although no specific study has been conducted. For this purpose, cell membrane microarrays were developed and the superoxide production evoked by the mitochondrial activity was assayed in the presence of specific inhibitors: rotenone, antimycin A and azide. Once the protocol was set up on cell membrane isolated from rat brain areas, the effect of six antimalarial drugs (atovaquone, quinidine, doxycycline, mefloquine, artemisinin, and tafenoquine) and two essential oils (*Rosmarinus officinalis* and *Origanum majoricum*) were evaluated in multiple human samples. The basal activity was different depending on the type of tissue, the liver, jejunum and adrenal gland being the ones with the highest amount of superoxide. The antimalarial drugs studied showed specific behavior according to the type of human tissue analyzed, with atovaquone and quinidine producing the highest percentage of superoxide formation, and doxycycline the lowest. In conclusion, the analysis of superoxide production evaluated in cell membranes of a collection of human tissues allowed for the characterization of the safety profile of these antimalarial drugs against toxicity mediated by oxidative stress.

## 1. Introduction

Reactive oxygen species (ROS) are chemical compounds formed upon incomplete oxygen reduction [1]. They are natural by-products of normal cellular activity and participate in cell signaling [2]. However, the disbalance between ROS production and the antioxidant defense system in the body causes the breakdown of cellular function and toxicity. This may occur due to an overproduction of ROS or a decrease in the antioxidant defense mechanism. There are several important sources of ROS. One example is a monoamine oxidase (MAO), an enzyme located in the outer mitochondrial membrane and whose activation leads to the formation of H_2_O_2_, which is responsible for oxidative damage in neurons [3,4]. Another one is NADPH oxidase, an enzyme with the capacity to transport electrons through the plasma membrane and generate superoxide. There are different isoforms of this enzyme expressed in the central nervous system, such as NOX2, NOX3, or NOX4. Likewise, the expression of this enzyme has also been demonstrated in the peripheral nervous system, where isoforms like NOX2, NOX3, NOX4 or NOX5 can be found on hepatocytes, spleen or pancreas [5].

**Figure 1:**
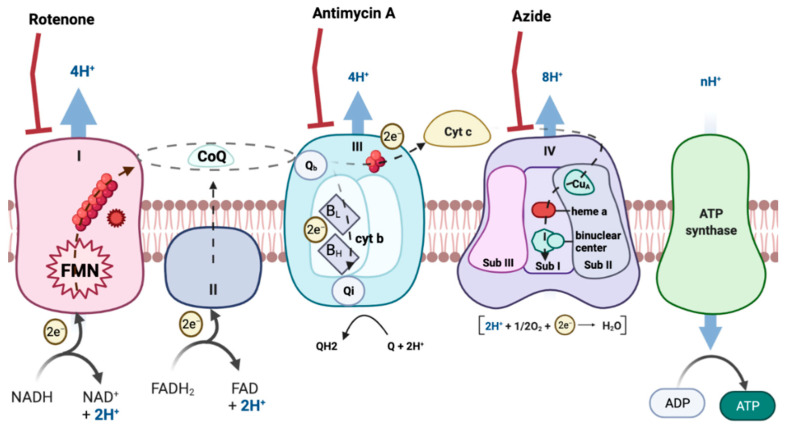
Electron transport chain. The image represents the different mitochondrial complexes, electron transporters and the electron flow. Created with BioRender.com.

ROS formation can occur in many tissues and through many routes. However, the mitochondrial respiratory chain (MRC) is one of the main producers of ROS [6] (Figure 1). MRC is located in the mitochondria, an organelle of most eukaryotic cells that carry out numerous biochemical reactions to maintain cellular physiology and homeostasis [7,8]. In this way, impaired function of mitochondria leads to damaged bioenergetics, calcium homeostasis dysregulation, decreased ATP production, increased production of free radicals, augmentation of oxidative stress and initiation of apoptotic processes [9,10].

With the development of new techniques for the study of respiratory complexes, new evidence has been discovered demonstrating a higher degree of organization of the different components of the respiratory chain called supercomplexes (SC). The composition and abundance of these SC can vary substantially depending on the organisms, tissue, metabolic and physiologic state [11,12,13,14,15], in addition to the lipid content of the mitochondrial inner membrane [16,17]. This suprastructure of the mitochondrial complexes is important because it decreases the distance for electron diffusion through the complexes, while increasing the efficiency of complexes I, III and IV [18,19]. This assembly regulates the electron flux in the MRC, optimizing the substrate oxidation [20] and limiting the production of ROS [21].

In physiological conditions ROS production is low and is controlled by antioxidant enzymes, such as superoxide dismutase and glutathione peroxidase [22]. However, under certain metabolic conditions or in presence of some compounds, ROS formation increases and triggers cellular stress. The main species that form ROS are superoxide anion, hydrogen peroxide and hydroxyl radical [23]. The formation of these radicals occurs mainly in complexes I and III of the mitochondrial transport chain, although other enzymes may also be involved [24]. Oxidative stress is one of the major causes of toxicity because the interaction of ROS with macromolecules and cellular structures interferes with different metabolic pathways, such as signaling pathways, as well as directly affecting cellular energy production [25]. Mitochondria compensate ROS by various cellular defense systems and antioxidants. Failure in this process can lead to overproduction of ROS, triggering pathologies such as cancer, cardiovascular and liver diseases, as well as neurodegenerative conditions [2]. In this regard, the main side effects of some antimalarials such as mefloquine, quinidine and tafenoquine are associated with cardio and hepatotoxicity [26,27,28]. In addition, several studies have shown that antimalarial drug artemisinin, as well as its analogs, produce cytotoxic effects in healthy human bronchial epithelium cells [29] and porcine oocytes during maturation in vitro assays [30]. Additionally, the relationship between the potential toxic effect produced by artesunate and the generation of ROS has been determined [31]. Furthermore, the antimalarial drug quinine produces a progressive decrease in the activity of the electron transport chain and a decrease in the formation of ATP in rat heart mitochondria [32]. This alteration of mitochondrial oxidative phosphorylation might be a mechanism of toxicity mediated by these drugs.

Malaria is an infectious disease caused by parasitic protozoa of the genus *Plasmodium* that destroy human erythrocytes. The global malaria burden in 2019 was about 229 millon new cases causing 409,000 deaths, over 90% of which ocurred in sub-Saharan African children under 5 years of age. Early diagnosis and treatment of malaria mitigate the incidence of the disease, reduce its deadly effects and help prevent its transmission. Pharmacological arsenal for prophylaxis and treatment of malaria is varied, although, the most common treatment is based in a combined therapy with artemisinin (or derivatives). Drug choice, or combination of drugs, is determined by the *Plasmodium* species, the clinical condition of the patient and the susceptibility of the infecting parasite according to the geographical area of infection and previous use of antimalarials [33]. However, most of the drugs used for the treatment of this disease present frequent side effects such as gastrointestinal disruption or neurotoxicity [34,35].

Natural products from plants are a potential source for the discovery of antimalarial drugs [36,37] and an alternative strategy for the treatment of malaria due to the emergence of antimalarial drug resistance [38] and frequent secondary effects of actual treatments. Essential oils (EOs) from aromatic plants have demonstrated antimalarial and antioxidant properties [39]. EOs with antimalarial properties have been obtained from *Hexalobus crispiflorus*, *Ripia multiflora* [40], *Ambrosia tenuifolia* [41], *Artemisia gorgonum* [42], *Xylopia* spp and *Artemisia annua* [43]. In this study we have evaluated the potential antimalarial activity of EOs from selected chemotypes of *Origanum majoricum* and *Rosmarinus officinalis* with reported antioxidant and antiparasitic properties [44,45,46,47].

Here, we present a biotechnological high-throughput tool based on cell membrane microarrays (CMMA) to study human tissue-specific superoxide production evoked by natural and synthetic drugs as well as essential oils, using nitro blue tetrazolium (NBT) as an indicator of superoxide formation.

## 2. Materials and Methods

### 2.1. Tissue Samples and Reagents

#### 2.1.1. Drugs and Reagents

Nitro blue tetrazolium (NBT), beta-nicotinamide adenine dinucleotide (NADH), decylubiquinone (dUQ), rotenone, antimycin A, sodium azide, atovaquone, quinidine, mefloquine hydrochloride, doxycycline hydrochloride, artemisinin and tafenoquine succinate were purchased from Sigma Aldrich (St-Louis, IL, USA).

#### 2.1.2. Samples

Sprague-Dawley rats (250–300 g) were purchased from Harlan (Barcelona, Spain) and maintained under standard conditions (food and water ad libitum; 12 h/12 h light/dark cycle, light on 7:00 a.m.) according to the institutional guidelines and Directive 86/609/EEC. After being deeply anesthetized with aketamine/xylazine cocktail (60 mg/kg of Ketamine-HCl and 5 mg/kg Xylazine- HCl i.p.), the rats were decapitated and the heart of each animal was quickly removed at 4 °C to avoid protein denaturation. Tissue samples were stored at −80 °C. Human biopsy tissues were supplied by the AMSBIO (Abingdon, Oxfordshire, UK) tissue bank according to its ethical protocols.

### 2.2. Cell Membrane Microarray Fabrication

Microarrays were composed of a collection of cell membrane homogenates isolated from rat brains (thalamus, cortex, cerebellum and hippocampus) or human tissues (liver, jejunum, renal medulla, renal cortex adrenal gland, white blood cells, myocardium, duodenum and spleen). Briefly, samples were homogenized using a Teflon-glass grinder (Heidolph RZR 2020) or a disperser (Ultra-Turrax^®^ T10 basic, IKA) in 20 volumes of homogenized buffer (1 mM EGTA, 3 mM MgCl_2_, and 50 mM Tris-HCl, pH 7.4) supplemented with 250 mM sucrose. The crude homogenate was subjected to a 1500 g centrifugation (AllegraTM X 22R centrifuge, Beckman Coulter) for 5 min at 4 °C, and the resultant supernatant was centrifuged at 18,000× *g* (Microfuge^®^ 22R centrifuge, Beckman Coulter) for 15 min (4 °C). The pellet was washed in 20 volumes of homogenized buffer and re-centrifuged under the same conditions. The tubes were finally decanted and the pellets were frozen at −80 °C, except for one aliquot, which was used to determine the protein concentration. Protein concentration was measured by the Bradford method [48] and adjusted to the required concentrations.

Membrane homogenates were resuspended in buffer and printed (4 nL per spot, 3–5 replicates per sample) onto glass slides using a non-contact microarrayer (Nanoplotter NP 2.1), placing three replicates of each sample (7 nL/spot), into preactivated glass microscope slides. The printing was carried out under controlled humidity (relative humidity 60%) at 4 °C. CMMA were stored at −20 °C until usage.

### 2.3. Determination of Superoxide Formation Mediated by NADH-Ubiquinone Oxidoreductase in CMMA

The NADH-ubiquinone oxidoreductase activity was assayed in the rat brain and a collection of different human tissues in CMMA. They were incubated in presence of NADH 0.1 mM and NBT 0.05 mg/mL in phosphate buffer (5 mM; pH 7.4) for 2 h and 30 min in the absence or in the presence of dUQ (50 µM) and increasing concentrations of MRC inhibitors, rotenone (from 0.00001 to 5000 nM), antimycin A (from 0.001 to 10 µM) and sodium azide (from 0.1 to 100 mM). Subsequently, they were washed for 10 min in phosphate buffer and rapidly immersed in distilled water. Once dried, the final image of CMMA was assembled with the software Adobe Photoshop CS5 (Adobe Systems Incorporated, Mountain View, CA, USA) and quantified with MAPIX software 7.3.1 (Innopsys, Carbonne, France).

### 2.4. Determination of Superoxide Formation Mediated by Antimalarials in CMMA

Microarrays were incubated with the reaction solution containing NADH 0.35 mM, NBT 0.05 mg/mL, testing drug 10 µM and dUQ 50 µM in phosphate buffer (50 mM; pH 7.4) for 25 min. Subsequently, they were washed for 5 min in phosphate buffer and rapidly immersed in distilled water. Once dried, the final image of CMMA was acquired with the software Adobe Photoshop CS5 (Adobe Systems Incorporated, Mountain View, CA, USA) and quantified with MAPIX software 7.3.1 (Innopsys, Carbonne, France).

### 2.5. Data Analysis and Normalization

Data handling and analysis was carried out using Excel and GraphPad software (version 9.0). Results were expressed as means of independent data points ± S.E.M. or S.D. In sake of clarity, biochemical data (Complex I activity and superoxide production) are presented as percentage of controls with or without the application of MRC inhibitors and antimalarials. Data were analysed using student’s *t*-test and ANOVA. * *p* < 0.05 was considered significant.

### 2.6. Plant Material and Cultivation

The plant species selected for the evaluation of antiphytomonas activity included a two aromatic plant species from the Spanish native flora (identified by Dr. Daniel Gómez, IPE-CSIC) cultivated for 8 years in an experimental field located at Ejea de los Caballeros, Spain (42°8′8.73″ N, 1°12′31.50″ W). A detailed description of the field and the cultivation parameters has been reported [49,50].

Aerial plant parts were collected at the flowering stage during the year 2018.

### 2.7. Essential Oil Extraction

Hydrodistillation was performed using a Clevenger-type apparatus with an extraction chamber, separated according to the method recommended by the European Pharmacopoeia [51]. Each extraction was carried out in triplicate with 100 g of dried aerial plant parts for 3 h. The EOs were obtained by phase separation, eliminating the aqueous extracts. The EOs were dried over anhydrous sodium sulfate and stored at 4–6 °C until the chemical analysis.

### 2.8. Gas Chromatography–Mass Spectrometry Analysis

Essential oils were analyzed by gas chromatography-mass spectrometry (GC-MS) using a Shimadzu GC-2010 gas chromatograph coupled to a Shimadzu GCMS-QP2010 Ultra mass detector (electron ionization, 70 eV, Duisburg, Germany) and equipped with a 30 m × 0.25 mm i.d. capillary column (0.25 µm film thickness) Teknokroma TRB-5 (95%) dimethyl-(5%) diphenylpolisiloxane. The working conditions were as follows: split ratio (20:1), injector temperature 300 °C, temperature of the transfer line connecter to the mass spectrometer 250 °C, initial column temperature 70 °C, then heated to 290 °C for 15 min. Electron ionization mass spectra and retention data were used to assess the identity of the compounds by comparing them with those of the standards or those found in the Wiley 275 Mass Spectral Database and NIST17. The relative amounts of individual components were calculated based on the GC peak area (flame ionization Detector, FID, response), without using a correction factor. The relative standard deviation (calculated as SD/average × 100) for the % peak area (three injections/sample) was ≤1%.

### 2.9. Ferriprotoporphyrin (FP) IX Biocrystallization Inhibition Test (FBIT)

This bioassay was performed to evaluate the inhibition of FP biocrystallization in presence of the essential oils as described [52]. The bioassay was carried out in a non-sterile 96-well plate flat-bottom at 37 °C for 24 h. The EOs were tested at 10, 5, 2.5, 1.25, 0.63 0.31 and 0.16 mg/mL; Chloroquine biphosphate (Sigma-Aldrich) was used as the reference drug at 0.1 mg/mL. The following solutions were added to the plate: 0.5 mg/mL of hemin chloride (Sigma-Aldrich) in dimethylsulphoxide (DMSO) (50 µL), 100 µL of 0.5 M sodium acetate buffer (pH 4.4), and 50 µL of EOs in DMSO. After 24 h, the plates were centrifuged at 3000 rpm for 5 min, and the supernatant was discarded. The remaining pellet was resuspended in 200 µL of DMSO so as to remove the unreacted FP. The plate was centrifuged again, and the supernatant was discarded. The pellet was dissolved in 150 µL of 0.1 M NaOH, and the absorbance measured at 405 nm. The percentage of inhibition of the FP biocrystallization was calculated as follows: Inhibition (%) = 100 × [(OD control − OD treatment)/OD control].

## 3. Results

### 3.1. Protocol Optimization to Detect Superoxide Formation in Cell Membrane Microarrays

**Figure 2:**
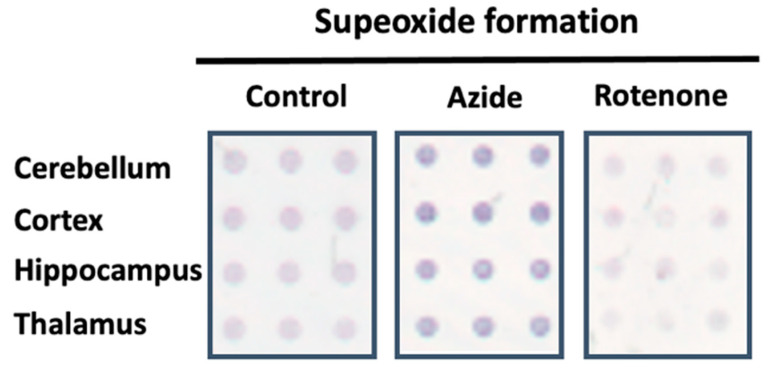
Representative image of the superoxide formation evoked by NADH dehydrogenase activity in absence (control) or in the presence of MRC inhibitors using CMMAs composed of different rat brain areas. The dark blue color symbolizes the reduced state of nitroblue tetrazolium (NBT), which indicates an increased formation of superoxide when the blue color is more intense.

To characterize the electron transport chain and demonstrate the capabilities of the methodology, CMMA containing three replicates of rat cerebellum, cortex, hippocampus and thalamus were printed. The arrays were used for superoxide formation determination following the protocol described in Section 2. The effect of different inhibitors, such as rotenone for the complex I, antimycin A for the complex III and azide for the complex IV on the mitochondrial transport chain was evaluated (Figure 2).

Firstly, enzymatic assays were performed to determine the basal activity of the electron transport chain in presence of decylubiquinone transporter (Figure S1). The assay results showed that the basal activity in superoxide formation varied among some of the tissues studied. In particular, statistically significant differences were observed between the cortex and the thalamus.

**Figure 3:**
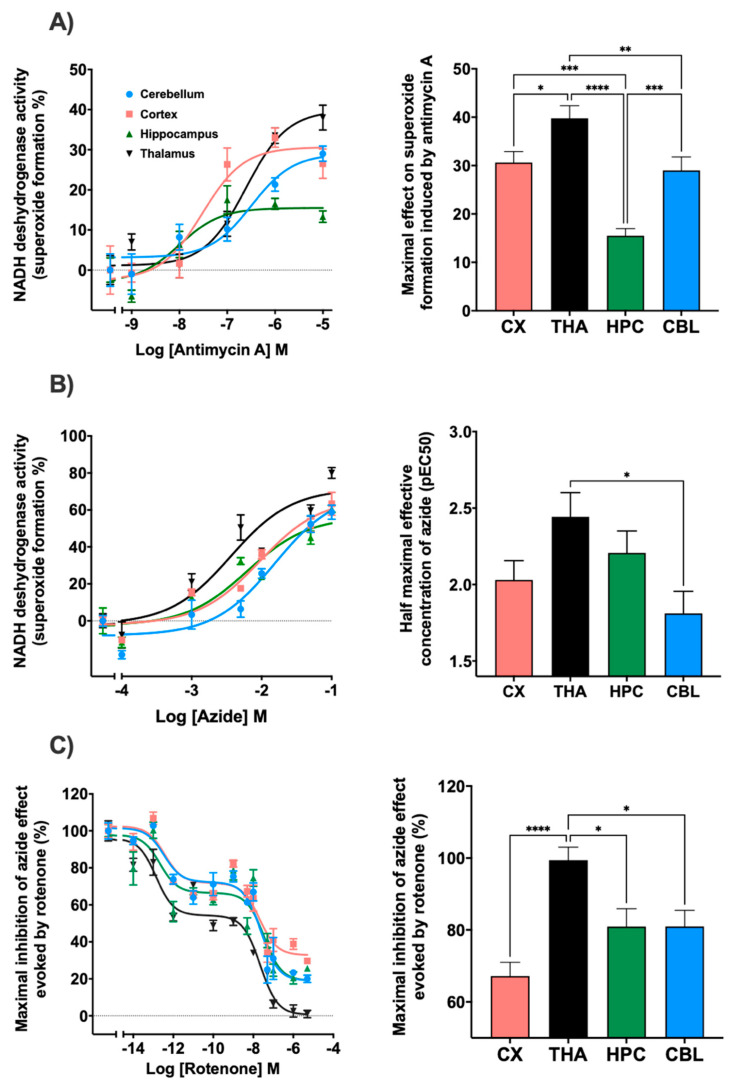
Different trend lines represent the NADH dehydrogenase activity expressed as percentages over basal activity in rat cortex, thalamus, hippocampus and cerebellum in the presence of decreasing concentrations of mitochondrial electron transport chain inhibitors. The histograms represent: (**A**) The maximal effect on superoxide formation induced by antimycin A. Thalamus showed significative differences with cortex (* *p* < 0.05), cerebellum (** *p* < 0.01) and hippocampus (**** *p* < 0.0001). Hippocampus also presented significative differences with cerebellum and cortex (*** *p* < 0.001); (**B**) Half maximal effective concentration (pEC50) of azide on superoxide formation; significative differences were observed between thalamus and cerebellum (* *p* < 0.05); (**C**) the inhibition of NADH dehydrogenase activity induced by rotenone in the presence of sodium azide. Thalamus showed significative differences with hippocampus, cerebellum (* *p* < 0.05) and cortex (**** *p* < 0.0001). Data are mean ± SEM values, *n* = 5.

In relation to the complex III inhibitor, antimycin A, regional differences were observed in terms of the maximum effect produced. In the thalamus, a maximum effect of 39.7% was reached, while in the cortex and cerebellum the values remained at 29.0% and 30.6%. In the hippocampus, the maximum effect reached was 15.5% (Figure 3A). Regarding the potency, the cortex presented significant differences with thalamus and cerebellum (Figure S2). Sodium azide inhibited mitochondrial complex IV with a similar result in all brain areas such as the cerebellum, cortex, and thalamus reaching values of 78%, 68.2%, and 73.3%, respectively. The hippocampus was the region where the lowest inhibitor effect was obtained with an impact of 58.6%. Significant differences were observed in the potency of this drug between the thalamus and cerebellum (Figure 3B). In addition, the inhibition of the mitochondrial complex I, NADH-ubiquinone oxidoreductase, by the action of rotenone was determined (Figure 3C). The maximal effect of superoxide formation was established by adding sodium azide without any inhibitor. When using azide in the presence of rotenone, a decrease in the maximal superoxide formation was observed with significant differences between the thalamus and the rest of the brain areas. Inhibition values of 81.0% in the cerebellum and hippocampus, 67.2% in the cortex, and 99.4% in the thalamus were achieved. Furthermore, rotenone also slowed down NADH consumption. A two-phases curve is obtained in all the brain areas. A first decay phase was observed at a rotenone concentration of 0.01 pM and a second one at 5 nM. The curves were adjusted to competition for two binding sites.

Afterwards, the logarithm of half-maximum inhibitory or effective concentration (pIC50 or pEC50) and maximum effect (Imax or Emax) parameters were calculated. On the one hand, a model of two binding sites was used for rotenone, and on the other hand, a model of a binding site for antimycin A and azide (Table 1).

### 3.2. Effect of Antimalarials on Superoxide Formation in Human Tissues

#### 3.2.1. Basal Superoxide Formation in Human Tissues

**Figure 4:**
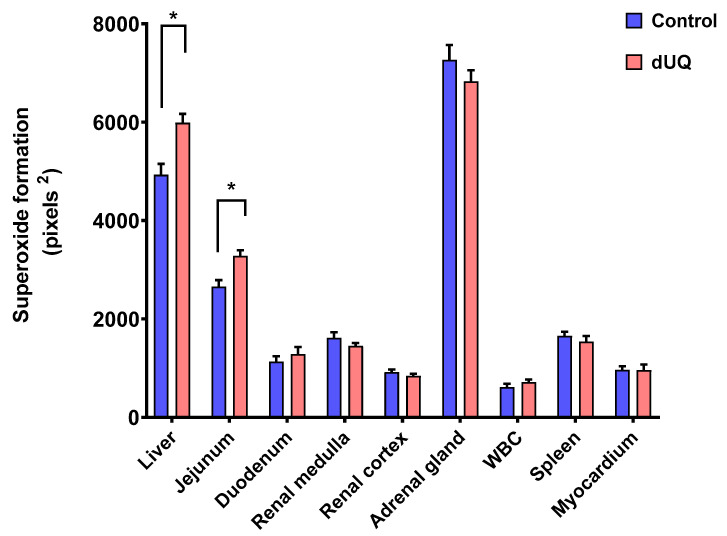
NADH-induced superoxide formation in cell membranes isolated from human tissues included in the microarray, in absence and presence of dUQ. Test t-student, * *p* < 0.05 dUQ vs control, *n* = 3.

NADH dehydrogenase activity was assessed by NADH-induced NBT reduction in nine different human tissues in absence and presence of dUQ. As shown in Figure 4, differences in basal superoxide formation behavior were observed between different analyzed tissues. Thus, while production of superoxides after adding dUQ increased in liver and jejunum, no variations were found in the rest of studied tissues.

#### 3.2.2. Superoxide Formation with Antimalarials in Human Tissues

Next, superoxide-forming capacity of the six most commonly used antimalarials was evaluated: Atovaquone, quinidine, mefloquine, doxycycline, artemisinin and tafenoquine. In general, drug-induced superoxide formation in absence and presence of dUQ showed a differential profile specific to the studied tissues. Thereby, cell membranes isolated from the liver, jejunum and adrenal gland showed a higher superoxide production.

**Figure 5:**
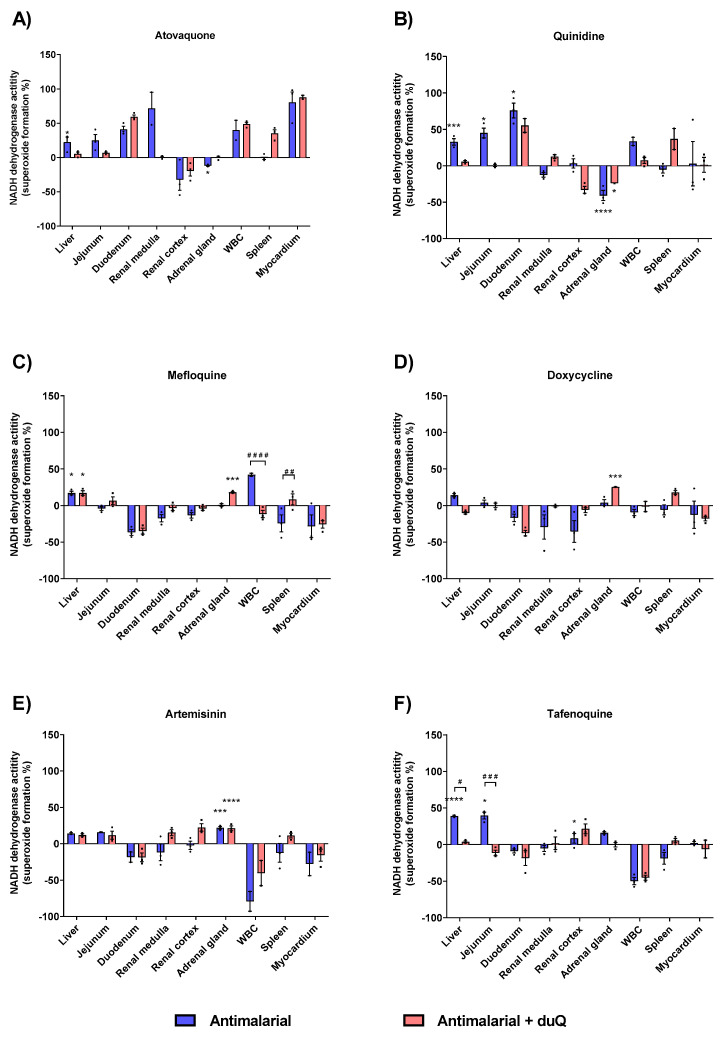
Superoxide production induced by action of antimalarials on MRC, expressed as percentages of stimulation over basal activity in absence of drug, in absence and presence of dUQ transporter. (**A**) Superoxide formation induced by atovaquone; (**B**) Superoxide formation induced by quinidine; (**C**) Superoxide formation induced by mefloquine; (**D**) Superoxide formation induced by doxycycline; (**E**) Superoxide formation induced by artemisin; (**F**) Superoxide formation induced by tafenoquine. ANOVA statistical test * *p* < 0.05; *** *p* < 0.001; **** *p* < 0.0001 antimalarial vs. control; # *p* < 0.05; ## *p* < 0.01; ### *p* < 0.001; #### *p* < 0.0001 antimalarial vs. antimalarial + dUQ. *n* = 3.

Atovaquone produced a higher superoxide formation compared to control in all tissues studied, except in renal cortex and adrenal gland. Out of the six antimalarials tested, atovaquone was the one with the highest percentage of superoxide formation, with a 80.3% (in absence of dUQ) and 87.9% (in presence of dUQ) in myocardium (Figure 5A). Attending to liver and adrenal gland, quinidine showed similar results to those obtained with the atovaquone. In liver, jejunum and duodenum superoxide formation was increased by quinidine while in adrenal gland a significant decrease was observed (Figure 5B). Mefloquine, as well as doxycycline, compared to atovaquone and quinidine, produced fewer superoxides. In fact, in tissues where there was an increase in superoxide formation, the maximum increase did not exceed 50% versus control (Figure 5C) for mefloquine and 25% versus control for doxycycline (Figure 5D). Artemisinin produced an increase of superoxide formation in all studied tissues except in duodenum, white blood cells and myocardium, where a decrease was observed (Figure 5E). The greatest increase of superoxide formation was in the adrenal gland, significant in absence and presence of dUQ.

Finally, tafenoquine increased superoxide formation in the liver, jejunum and adrenal gland (Figure 5F). In presence of dUQ variations were found among studied tissues, decreasing tafenoquine’s effect in liver and jejunum.

### 3.3. Composition, Antimalarial Activity and Effect on Superoxide Formation of Essential Oils

#### 3.3.1. Antimalarial Activity of EOs

**Figure 6:**
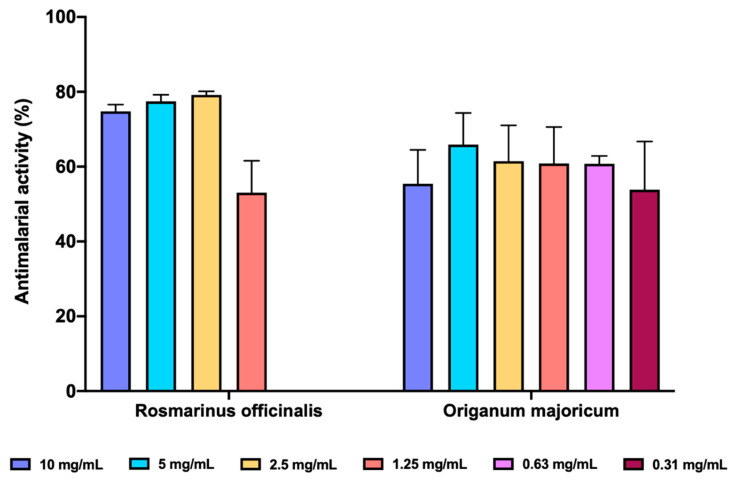
In vitro antimalarial activity of *R. officinalis* and *O. majoricum* EOs. Data are expressed as percentage of inhibition ± standard deviation (SD). All assays were carried out in quadruplicate.

We determined the inhibition of FP biocrystallization as a surrogate antimalarial activity mediated by inhibition of heme polymerization. Both *O. majoricum* and *R. officinalis* presented potencial antimalarial activity (Figure 6), been R. officinalis the most effective at the three highest concentrations tested (10, 5 and 2.5 mg/mL) with a percentage of inhibition between 74.81–79.18%, whilst O. majoricum was the EO with activity at the lowest concentration (0.31 mg/mL). Cloroquine was used as a reference drug at 0.1 mg/mL with an activity of 73.83 ± 7.65%.

#### 3.3.2. Chemical Composition of EOs

The chemical analysis of the tested EOs is shown in Table 2. *R. officinalis* EO had 1,8-cineole (26%), α-pinene (21%) and camphor (13%) as major components, whereas *O. majoricum* EO contained 4-terpineol (30%) and γ-terpinene (11%).

#### 3.3.3. Effect on Superoxide Formation of EOs

In order to determine the effect of natural compounds on the superoxide forming capacity induced by NADH dehydrogenase, enzymatic assays were carried out using two natural extracts. The natural oils, *O. majoricun* and *R. officinalis*, were employed in experiments with human CMMA. The effect of the natural oils on superoxide formation was studied in the presence and absence of the mitochondrial transporter dUQ following the same methodology as in the previous section. The results showed that the essential oils favor the superoxide formation in a tissue-specific manner.

**Figure 7:**
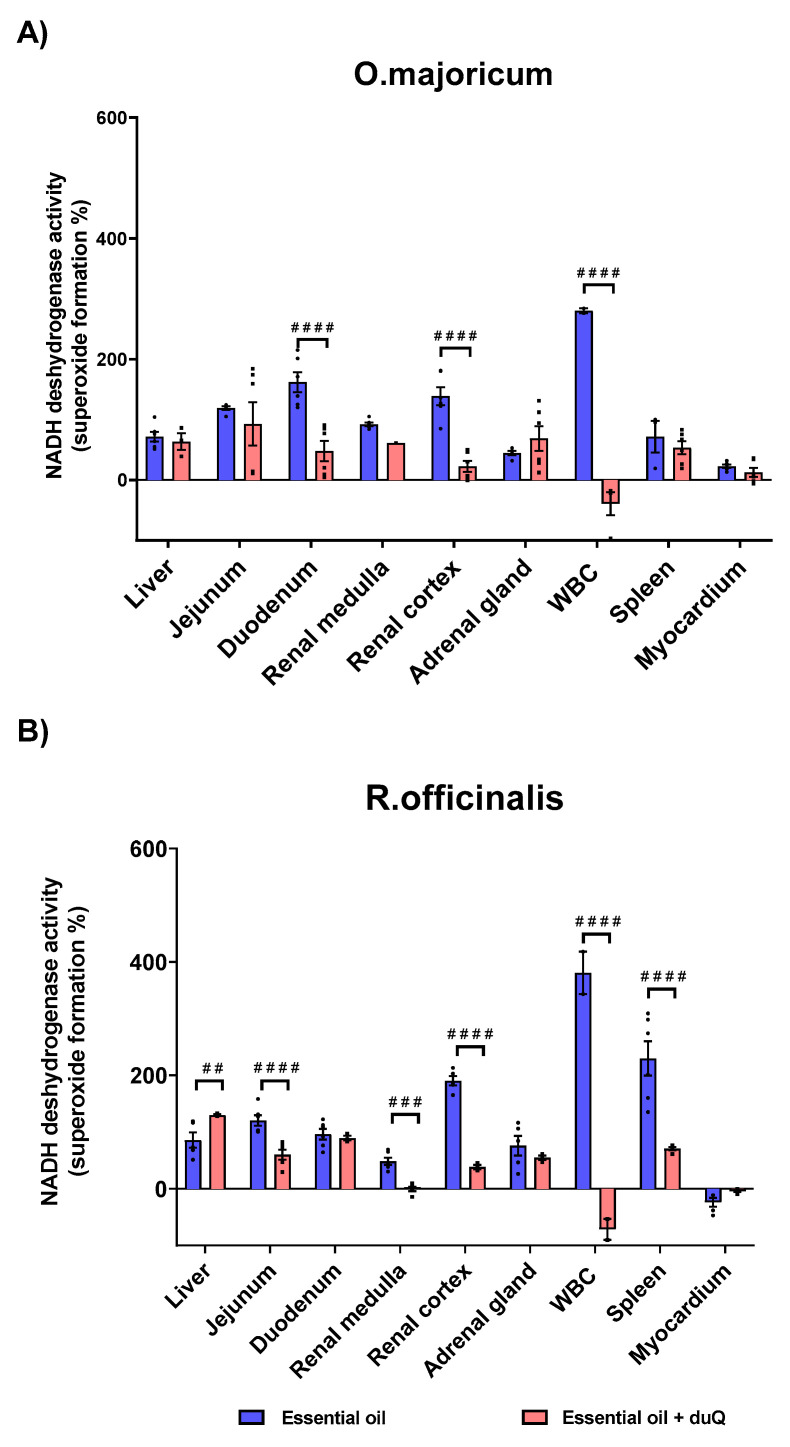
Superoxide production induced by the action of *O. majoricum* and *R. officinalis*, expressed as percentages of stimulation over basal activity in absence of drug, in absence and presence of dUQ transporter. (**A**) Superoxide formation induced by *O. majoricum*; (**B**) Superoxide formation induced by *R. officinalis*. ## *p* < 0.01; ### *p* < 0.001; #### *p* < 0.0001 essential oil vs. essential + dUQ. Data are mean ± SEM values, *n* = 3.

White blood cell (WBC) was the most sensitive tissue to essential oils in terms of superoxide formation. The increase was 280% for *O.majoricum* and 380.5% for *R. officinalis*. In the presence of the dUQ transporter, the percentage of superoxide formation was significantly reduced, reaching values below basal. Regarding to *O.majoricum*, an increase on superoxide formation was observed in all tissues studied except in the myocardium (Table S1). In the presence of the mitochondrial transporter, oxidative stress was significantly decreased in tissues such as the duodenum and renal cortex (Figure 7A). *R. officinalis* essential oil also increased superoxide production in all studied tissues except myocardium (Table S1). In the presence of dUQ, superoxide formation increased in liver, while in tissues such as the jejunum, renal cortex and spleen, superoxide production significantly decreased. In addition, in renal medulla the values were lower than basal (Figure 7B).

## 4. Discussion

This study has described the application of cell membrane microarrays to determine the specific actions of antimalarial drugs on human tissues by monitoring the production of superoxide, one of the main ROS, as indicator of celular stress. For this purpose, firstly, the experimental protocol was validated using CMMA from a set of rat brain areas, whose stability and functionality has already been confirmed by different methods, together with specific inhibitors of the oxidative phosphorylation [53,54,55,56,57].

The superoxide production evoked by NADH dehydrogenases was higher in cerebral cortex following by hippocampus, cerebellum and thalamus. In this line, a higher activity of the complex I was reported in brain cortex homogenates than in cerebellum of young mice, while it was not observed in aged brain [58] The tissue-specific expression of complex I determined in vivo by [^3^H]DHR binding correlates in vitro with activities of complex II and complex IV, suggesting that the stoichiometry of the mitochondrial complexes of the electron transport chain is relatively constant across brain areas [59].However, tissue-specific isozymes could determine regional features of these mitochondrial respiratory complexes [60].

The superoxide production evoked in presence of the specific inhibitors of mitochondrial respiration was different depending on the brain region studied. The mitochondrial complex III inhibitor, antimycin A, increased superoxide formation with a different rank order for the maximal effect (thalamus > cerebral cortex > cerebellum > hippocampus) and for the potency (hippocampus > cerebral cortex > thalamus > cerebellum). This discrepancy between the maximal effect (cerebellum > thalamus > cerebral cortex > hippocampus) and the potency (thalamus > hippocampus > cerebral cortex > cerebellum) among brain areas was also observed with the mitochondrial complex IV inhibitor, azide. In this sense, specific differences in the activities of complex III and IV have been described in human cerebellum, frontal cortex and hippocampus [61]. These differences could be due to the functional and molecular diversity of this organulle in each brain area [62]. In addition to its homeostatic role in preserving essential functions, cell-type specific molecular diversity of mitochondria play a important function in the healthy brain and its selective vulnerability during disease [62,63] as well as in drugs sensitivity and side-effects [64,65].

This diversity might be an explanation of why some brain areas are specially affected in neurodegenerative processes, as happens, for example, with the dopaminergic neurons of the *substantia nigra* in Parkinson’s Disease. These neurons are particularly sensitive to a neurotoxin, MPTP (1-methyl-4-phenyl-1,2,3,6-tetrahydropyridine), which active metabolite MPP is an inhibitor of mitochondrial complex I. I In monkeys treated with this drug, it has been seen that the most affected pathway is the nigrostriatal, including greater cell loss in *substancia nigra* and ventral tegmental area [66]. In other animal models of the disease, the locus coeruleus and the substantia nigra compacta show higher levels of mitochondrial stress than other areas as the motor cortex or hippocampus [67,68]. These results fit with the progressive pattern of neurodegeneration observed in this disease [69]. Furthermore, in other diseases like depression, oxidative stress and free radicals play a fundamental role in the progression of the disease [70,71].

Mitochondrial complex I, III and IV can be associated to form different supramolecular structures known as SC [14,72,73]. This assembly modulates the electron flux in the mitochondrial electron transport optimizing the use of available substrates [20] and determining the production of ROS [21]. In this context, the enhancement of the superoxide production evoked by azide in all the brain areas included in CMMA was blocked by the inhibition of the mitocochondrial complex I activity by rotenone, according to a biphasic model, with a pIC50 in the picomolar range for the high affinity binding site and in the micromolar range for the low affinity binding site. No differences were identified in the potency of rotenone neither in the pIC50 high nor pIC50 low among brain areas. However, statistically significant changes were observed in relation to the maximum inhibitory effect evoked by rotenone (thalamus > cerebellum = hippocampus > cerebral cortex). In this sense, autoradiographic studies using [^3^H]-dihydrorotenone has demonstrated not only a specific profile of complex I distribution in rat brain but also that NADH enhances binding 4- to 80-fold (Kd) by increasing the density of the active conformation of the complex I [74], which is dependent on interaction with other mitochondrial complexes [75,76,77]. Thus, the two populations of rotenone-sensitive activities observed after the inhibition of complex IV could indicate a different composition or functional state of supercomplex assembly in full-associated or weakly-associated respirasomes [78], althought further specific investigation must be performed to validate this hypothesis.

Once the protocol for determining superoxide production by rat CMMA was establised, the effects of six antimalarial drugs (atovaquone, quinidine, doxycycline, mefloquine, arte-misinin, and tafenoquine) and two essential oils (*Rosmarinus officinalis* and *Origanum majoricum*) were evaluated using human CMMA in nine human tissues and organs, including multiple body systems such as the digestive, cardiac, renal and immune systems. The superoxide production evoked by NADH dehydrogenase activity was different depeninding on the tissue analysed, being liver and adrenal gland the ones with the highest basal rate, both of them tissues with abundant mitochondria [79]. This tissue-specific profile was also observed in presence of the electron carrier dUQ, which only promoted a significant increase of superoxide production in the liver and jejunum. In this regard, PET studies described a different density of mitochondrial complex I binding sites not only among rat brain areas, but also among peripheral tissues [80] Moreover, exogenous dUQ can be efficiently taken up by the liver, but not by other tissues like heart [24]. This feature regarding the accumulation of dUQ may be involved in the increased effect on superoxide production observed in the human liver.

Atovaquone, a naphthoquinone structurally analogous to dUQ, acts in mitochondria by inhibiting complex III of the MRC of Plasmodium falciparum causing alterations in membrane potential [81]. Renal adverse effects have not been reported for this drug but it is known to frequently provoke gastrointestinal adverse effects and elevation of liver enzymes [34]. In this regard, atovaquone did not alter the production of superoxide in the renal cortex and adrenal gland, but triggered the highest rate of superoxide production in human tissues from digestive system(jejunum, duodenumand liver). Quinidine presented a similar profile to atovaquone except in renal medulla, showing a significantly higher rate of superoxide formation in the liver, jejunum and duodenum. This compound has been described to produce gastrointestinal side effects including hepatotoxicity, diarrhea, digestive distress, nausea and vomiting, esophagitis [82]. Of note, quinidine did not increase the superoxide formation neither in renal, where it significantly reduced it, nor in cardiactissue. The main antiarrhythmic actions of this molecule are attributted to the inhibition of the fast inward sodium currentand the potassium efflux during repolarization, thereby promoting a progressive decrease in MRC activity and ATP formation in heart mitochondria [32,82].

Mefloquine increased superoxide formation only in liver and WBC in absence of dUQ, while in the presence of this electron carrier the superoxide production was also incremented in adrenal gland and spleen. During malaria infection, mefloquine has been shown to induce apoptosis mediated by superoxide formation in the parasite as well as destabilization of the mitochondrial membrane potential, activation of caspases and inhibition of *Plasmodium* heme detoxification leading to the parasite’s death [19]. The cytotoxic capacity of Mefloquine has also been reported in cervical cancer cells where it inhibited mitochondrial function via blocking mitochondrial respiration, decreasing membrane potential, increasing ROS formation and decreasing ATP production [83].

Doxycycline was shown to promote low superoxide formation in the nine tissues analysed, being only significant in adrenal gland in combination with dUQ. These modest actions observed on digestive tissues contrast with the numerous gastrointestinal adverse effects described for this antibiotic and antimalarial drug [84], suggesting the implication of mechanisms other than the one studied in this work. In the same way, artemisinin evoked a slight increase in superoxide formation in liver, jejunum, and adrenal gland, that was also observed in presence of dUQ, not only in these tissues but also in kidney and spleen. By contrast, artemisinin reduced the superoxide formation in WBC and myocardium. In this sense, it has not been described in cases of cardiotoxicity in humans although have been reported in animals [35]. Tafenoquine specially increased the superoxide formation in liver and jejunum, in accordance with the gastrointestinal side effects (diarrhea, nausea and vomiting) described for this antimalarial drug [85]. This enhancement was abolished in both tissues by the electron carrier dUQ, probably by a competition for the ubiquinone binding site, similar to the mechanism of action of the type A inhibitors of mitochondrial complex I [86]. Thus, coadministration of CoQ during treatment with tafenoquine might contribute to mitigate the side effects derived from these tissues. The essential oils, *R. officinallis* and *O. majoricum*, evoked an increase in superoxide formation in several of the tissues tested, being especially higher in white blood cells, where the electron carrier dUQ totally inhibits this effect. This blockade, as with tafenoquine, suggests that dUQ competes with the active compounds of the EOs for binding sites at the site of action of NADH dehydrogenase enzymes. The complex composition of EOs makes it difficult to determine whether compounds with antimalarial properties are also responsible for the formation of tissue-specific ROS or whether it is due to other compounds present in the oils. Further studies should therefore be conducted to identify antimalarial compounds that produce fewer reactive oxygen species. In this way, a safer profile could be achieved, reducing the toxicity and side effects produced by superoxides.

In conclusion, the analysis of superoxide production determined in human tissue CMMA can provide relevant data on the oxidative stress generated in specific human tissues by antimalarial drugs. Thus, this technology could be a suitable tool to complement toxicity studies by assessing the vulnerability of a human tissue to a compound by quantifying oxidative stress in the preclinical phases of drug discovery.

## Figures and Tables

**Table 1:** The logarithm of half the maximum inhibitory or effective concentration (pIC50 or pEC50) and the maximum effect (Imax or Emax) was calculated for each inhibitor.

		Cerebellum	Cortex	Hippocampus	Thalamus
	pIC50 Hi	−12.4 ± 0.4	−12.4 ± 0.3	−12.6 ± 0.3	−12.8 ± 0.2
Rotenone	pIC50 Low	−7.6 ± 0.1	−7.6 ± 0.2	−7.4 ± 0.2	−7.6 ± 0.1
	Imax	81.0 ± 4.5	67.2 ± 3.8	81.0 ± 4.9	99.4 ± 3.6
Antimycin A	pEC50	−6.4 ± 0.2	−7.5 ± 0.2	−8.0 ± 0.2	−6.6 ± 0.1
	Emax	29.0 ± 2.7	30.6 ± 2,7	15.5 ± 1.4	39.7 ± 2.5
Azide	pEC50	−1.8 ± 0.1	−2.0 ± 0.1	−2.2 ± 0.1	−2.4 ± 0.1
	Emax	78.0 ± 6.6	68.2 ± 5.0	58.6 ± 5.1	73.3 ± 6.8

**Table 2:** Main components of *R. officinalis* and *O. majoricum* EOs.

EO	Main Components
*Rosmarinus officinalis*	1,8-cineol (26%), *α*-pinene (21%), camphor (13%), camphene (9%), *β*-pinene (5%),
*Origanum majoricum*	4-terpineol (30%), *γ*-terpinene (11%), α-terpinene (6%), sabinene (6%), *p*-cymene (5%), *trans-β*-ocimene (5%), caryophyllene (5%)

## Data Availability

The data support the findings of this study are available from the corresponding author, Gabriel Barreda-Gómez, upon reasonable request.

## Supplementary Materials

The following are available online at https://www.mdpi.com/article/10.3390/membranes11120943/s1, Figure S1: The histogram represent the basal activity on superoxide formation mediated by NADH dehydrogenase in presence of decylubiquinone transporter. CMMA used in the assay consisted of rat membrane homogenates isolated from cortex, thalamus, hippocampus and cerebellum. Significant differences between cortex and thalamus were observed (* *p* < 0.05). All the data are mean ± SEM values, Figure S2: The histogram represent the half maximal effective concentration of antimycin A (pEC50) on superoxide formation carried out in CMMAs consisting of rat membrane homogenates isolated from cortex, thalamus, hippocampus and cerebellum spotted in triplicate. The cortex showed significant differences with thalamus and cerebellum (* *p* < 0.05). All de data are mean ± SEM values, Table S1: Superoxide production induced by the action of essential oils carried out in CMMAs consisting of human membrane homogenates isolated from liver, jejunum, duodenum, renal medulla, renal cortex, adrenal gland, white blood cell (WBC), spleen and myocardium. Data was expressed as percentages of stimulation over basal activity. The results showed significant differences in tissues such as liver, jejunum, duodenum, renal medulla, renal cortex, adrenal gland, WBC and spleen (** *p* < 0.01, **** *p* < 0.0001). All de data are mean ± SEM values.
