# Supplementary material for: Study of Tissue-Specific Reactive Oxygen Species Formation by Cell Membrane Microarrays for the Characterization of Bioactive Compounds
Source: Membranes (Basel). 2021 Nov 29;11(12):943. doi: 10.3390/membranes11120943 (PMC8705675; doi:10.3390/membranes11120943)
Supplement: Supplementary file 1 [file membranes-11-00943-s001.zip › membranes-1466327-supplementary.pdf]

Supporting Information

Study of Tissue-Specific Reactive Oxygen Species Formation by Cell Membrane Microarrays for the Characterization of Bioactive Compounds

Ane Elexpe, Nerea Nieto, Claudia Fernández-Cuétara, Celtia Domínguez-Fernández, Teresa Morera-Herreras, María Torrecilla, Cristina Miguélez, Antonio Laso, Eneko Ochoa, María Bailen, Azucena González-Coloma, Iñigo Angulo-Barturen, Egoitz Astigarraga and Gabriel Barreda-Gómez

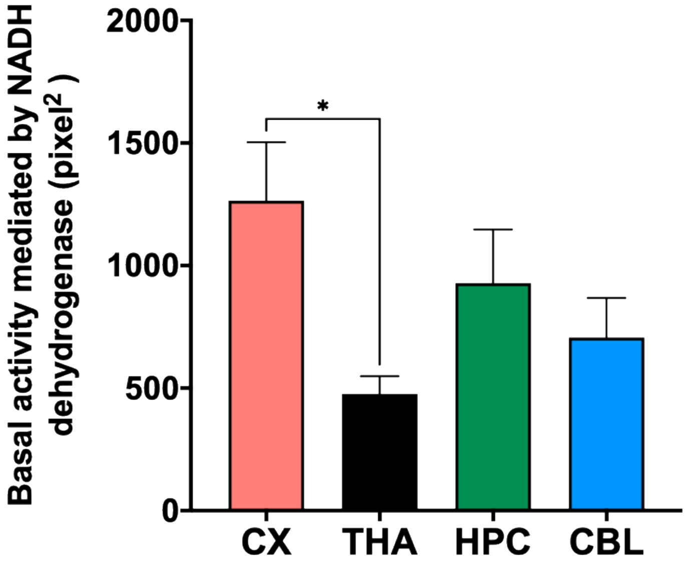

Figure S1. The histogram represent the basal activity on superoxide formation mediated by NADH dehydrogenase in presence of decylubiquinone transporter. CMMA used in the assay consisted of rat membrane homogenates isolated from cortex, thalamus, hippocampus and cerebellum. Significant differences between cortex and thalamus were observed (*$p < 0.05$). All the data are mean ± SEM vales.

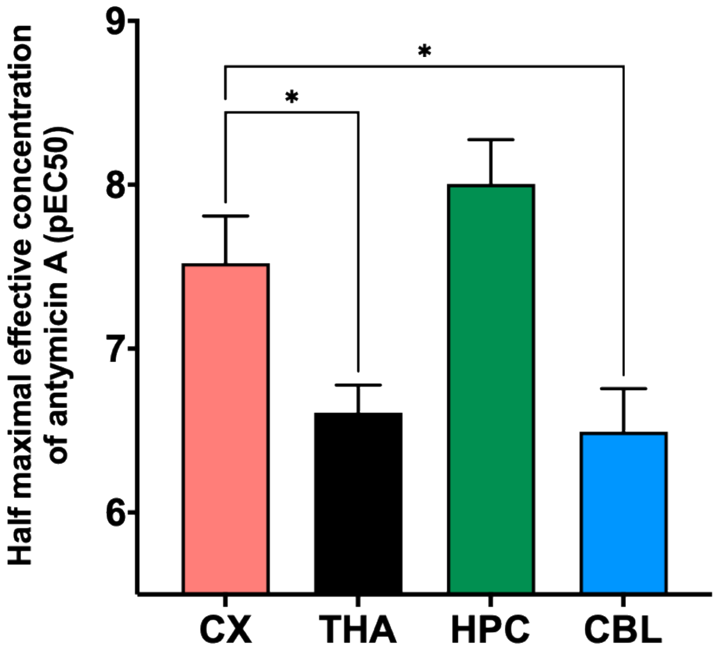

Figure S2. The histogram represent the half maximal effective concentration of antimycin A (pEC50) on superoxide formation carried out in CMMAs consisting of rat membrane homogenates isolated from cortex, thalamus, hippocampus and cerebellum spotted in triplicate. The cortex showed significant differences with thalamus and cerebellum (*$p < 0.05$). All de data are mean ± SEM values.

Table S1. Superoxide production induced by the action of essential oils carried out in CMMAs consisting of human membrane homogenates isolated from liver, jejunum, duodenum, renal medulla, renal cortex, adrenal gland, white blood cell (WBC), spleen and myocardium. Data was expressed as percentages of stimulation over basal activity. The results showed significant differences in tissues such as liver, jejunum, duodenum, renal medulla, renal cortex, adrenal gland, WBC and spleen (**$p < 0.01$, ****$p < 0.0001$). All de data are mean ± SEM values.

	O. Majoricum (% stm)	*R. Officinalis (% stm)*
Liver	71.7 ± 8.0 ****	85.4 ± 13.6 ****
Jejunum	119.0 ± 3.0 ****	120.0 ± 9.4 ****
Duodenum	161.8 ± 16.4 ****	95.8 ± 9.2 ****
Renal medulla	92.0 ± 3.1 ****	47.8 ± 6.4 ****
Renal cortex	138.5 ± 15.1 ****	190.2 ± 8.3 ****
Adrenal gland	44.6 ± 3.5 **	75.6 ± 17.3 ****
WBC	280.0 ± 4.0 ****	380.5 ± 37.5 ****
Spleen	71.3 ± 26.2 ****	229.5 ± 30.3 ****
Myocardium	22.5 ± 2.8	−23.6 ± 7.8
